# Meta-analysis of RNA-Seq studies reveals genes with dominant functions during flower bud endo- to eco-dormancy transition in *Prunus* species

**DOI:** 10.1038/s41598-021-92600-6

**Published:** 2021-06-23

**Authors:** Monica Canton, Cristian Forestan, Claudio Bonghi, Serena Varotto

**Affiliations:** 1 Department of Agriculture, Food, Natural resources, Animals and Environment (DAFNAE), University of Padua, Agripolis, Viale dell’Università, 16, 35020 Legnaro, PD Italy; 2 Present Address: Department of Agricultural and Food Sciences (DISTAL), University of Bologna, Viale Fanin 44, 40127 Bologna, Italy

**Keywords:** Computational biology and bioinformatics, Developmental biology, Plant sciences

## Abstract

In deciduous fruit trees, entrance into dormancy occurs in later summer/fall, concomitantly with the shortening of day length and decrease in temperature. Dormancy can be divided into endodormancy, ecodormancy and paradormancy. In *Prunus* species flower buds, entrance into the dormant stage occurs when the apical meristem is partially differentiated; during dormancy, flower verticils continue their growth and differentiation. Each species and/or cultivar requires exposure to low winter temperature followed by warm temperatures, quantified as chilling and heat requirements, to remove the physiological blocks that inhibit budburst. A comprehensive meta-analysis of transcriptomic studies on flower buds of sweet cherry, apricot and peach was conducted, by investigating the gene expression profiles during bud endo- to ecodormancy transition in genotypes differing in chilling requirements. Conserved and distinctive expression patterns were observed, allowing the identification of gene specifically associated with endodormancy or ecodormancy. In addition to the MADS-box transcription factor family, hormone-related genes, chromatin modifiers, macro- and micro-gametogenesis related genes and environmental integrators, were identified as novel biomarker candidates for flower bud development during winter in stone fruits. In parallel, flower bud differentiation processes were associated to dormancy progression and termination and to environmental factors triggering dormancy phase-specific gene expression.

## Introduction

The vegetative and reproductive meristems of many perennial species adapted to temperate zones survive the low temperatures period in a quiescent stage within buds, through a biological process named dormancy^1^. In deciduous fruit trees, entrance into the dormant state happens in late summer/fall, in correspondence to a shortening day length and decrease in temperature^2^. In winter, buds were originally considered to be in a resting state and thus this state was termed “dormancy.” Indeed, dormancy can be defined as “a state of self-arrest of the shoot apical meristem (SAM) which is maintained under growth promoting conditions”^3^. Lang et al.^4^ proposed that dormancy in temperate trees can be divided in three phases: endodormancy, ecodormancy and paradormancy, when growth inhibition is driven by the dormant bud itself, environmental conditions, or by other structures of the same plant, respectively. In *Prunus* species, this definition can be applied to vegetative buds in which the growth cessation of a completely differentiated apical meristem is clearly visible and the resumption of growth results in the formation of a shoot. By contrast, in flower buds entrance into the dormant stage occurs when the apical meristem is partially differentiated and during dormancy verticils continue growth and differentiation^5^. The continuous growth and differentiation of dormant *Prunus* flower buds is testified to by change of transcriptome landscape, metabolic activities including starch accumulation and hormone fluctuations, and ongoing floral structure differentiation^6^. In *Prunus*, each species and/or cultivar requires exposure to low winter temperature followed by warm temperatures, quantified as chilling requirements (CR) and heat requirements (HR) respectively, in order to remove the physiological blocks that inhibit budburst^7^. CR is estimated by measuring the number of chilling units or chilling portion accumulated at temperatures below a threshold value, commonly assumed to be between 6.8 and 7.2°C^8,9^; while HR is calculated by using the Growing Degree Hours or Growing Degree Days model^4^. After endodormancy and the fulfillment of CR, in the ecodormancy phase flower buds achieve full developmental capacity and resume growth after a certain amount of warmth (HR), which is strictly genotype dependent^10^. It has been reported that the transition from endodormancy to ecodormancy is an irreversible process, however, there is no univocal bud phenotype that identifies the physiological property of endodormancy other than cytological observations, measurements of chill, studies of starch accumulation in sweet cherry floral buds accumulation^7^ and expression of genes involved in the sporopollein biosynthesis^11^.

At genetic level, CR is a quantitative trait with a major effect on flowering time in all *Prunus* spp. in which it has been studied^12^. In peach, an association study conducted with four hundred different genotypes having different CR demonstrated a strong association between a previously identified QTL on chromosome 1 and CR^13^. This QTL in LG1 contains six tandemly repeats DORMANCY ASSOCIATED MADS-box (DAM 1–6) transcription factors. Leida et al.^14^ indicated evidence of epigenetic regulation over DAM genes in peach. They observed variation in chromatin marks at DAM6 locus i.e., decrease of H3K4me3 and increase of H3K27me3 at the promoter, TSS and largest intron of the gene. A decrease in the acetylation levels of H3 (H3ac) at DAM 6 loci was also detected at dormancy release. In addition, a significant enrichment of H3K27me3 was revealed at specific regions of this locus during dormancy release^1^, which could contribute to transcriptional downregulation observed for peach DAM genes during dormancy.

In addition to DAMs, other transcription factors and regulatory genes have been demonstrated to play an important role in dormancy. CBF genes play an integral role in the induction of freezing tolerance of both herbaceous and woody plants^15^. Additionally, CBF expression can alter parameters other than freezing tolerance, such as growth and the timing and development of flowering^16^.

At physiological level, Abscisic Acid (ABA) represents a key hormone in the regulation of flower bud dormancy in *Prunus* spp. Its content decreases during the process of peach flower bud endodormancy (corresponding to fulfillment of CR) and reaches a minimum level during the ecodormancy stage^17^. ABA has been hypothesized to control dormancy cycle by inhibiting cell proliferation and growth, antagonizing other hormones, such as gibberellins (GAs) and cytokinin. However, the role of ABA and GAs and other hormones in dormancy is based on their general defined function: whether ABA and GAs directly establish and unlock dormancy still needs further studies.

In order to gain new insights into the timing and progression of bud dormancy in *Prunus* species, we conducted a comprehensive meta-analysis of transcriptomic studies on flower buds of sweet cherry, apricot and peach. In this work, we re-analyzed and interpreted three RNA-Seq data experiments for the identification of: (1) common regulatory genes of endodormancy in addition to DAMs, 2) genes involved in the transition between endo and ecodormancy. The identification of gene markers able to quantify the dormancy status of the bud is a mandatory step because studies on dormancy break still need to be based on observations of growth onset in chilling–forcing experiments at the whole plant level. Markers have been searched amongst genes i) controlling floral bud differentiation, ii) involved in hormone metabolism and actions, iii) related to adaptation to environmental factors. Particular attention has been paid to regulators of transcription via epigenetic mechanisms.

All this information will help to decipher the key steps of the dormancy cycle in *Prunus* spp., with the final goal being to characterize fruit trees cultivars and breed new varieties better adapted to future scenarios of climate change.

## Results and discussion

### Meta-analysis of publicly available RNA-Seq data reveals differentially expressed genes (DEGs) during endo- to eco-dormancy transition in *Prunus* species

To investigate the transcriptional landscapes of dormant flower buds in *Prunus* species, we downloaded and analyzed publicly available RNA-Seq data of peach, apricot and sweet cherry^18,19^. The three selected studies include, for each species, varieties differing in chilling requirement, hereinafter called low (LCR) and high (HCR) chilling requirement (Supplemental Figs. 2, 3 and 4). RNA-Seq data were processed using a standard pipeline including removal of low-quality reads, aligning to the specie-specific reference genome and counting reads. Differently from original papers, high quality reference genome sequence and annotation is now available also for *P. armeniaca* and *P. avium*.

After assessing the structure of the dataset for each species, samples corresponding to endodormancy entrance (En1), progression (En2) and ecodormancy (Eco) stages were identified (Supplemental Tables 1–3; Supplemental Figs. 2–4). We therefore conducted differential gene expression analysis separately on each species, applying the Likelihood ratio test (LRT) with DESeq2. For each species, three time-course differential expression analyses were performed to identify significantly differentially expressed genes (DEGs) during endodormancy release in: i) HCR genotypes, ii) LCR genotypes or iii) between HCR and LCR genotypes (Genotype x Dormancy interaction). The results of differential expression tests are summarized in Table 1 and Supplemental Figs. 5a, 6a and 7a; the full lists of DEGs and their expression values are instead reported in Supplemental Datasets 1–3.

**Table 1:** Number of differentially expressed genes (DEGs) identified in each LRT test for each species, together with the number of non-redundant DEGs in each species.

Species	test	# DEGs
*P. persica*		9586
	HCR	5912
	LCR	7048
	G × D Interaction	2664
*P. armeniaca*		8621
	HCR	4871
	LCR	5319
	Interaction	3341
*P. avium*		8072
	HCR	4348
	LCR	2633
	Interaction	4667

The time-series transcriptome profiles in peach, apricot and sweet cherry flower buds allowed us both to study the gene expression changes as bud development proceeds during dormancy, and to compare the expression data from three Rosaceae related species and between genotypes varying in chilling requirement.

To investigate the biological processes regulated during endodormancy release, we therefore evaluated Mapman functional enrichment patterns for the differentially expressed genes identified in HCR and LCR genotypes of the three species (Supplemental Figs. 5b, 6b and 7b). At first glance functional categories related to “cell cycle organization”, “external stimuli response”, “transcriptional regulation”, “phytohormone action”, “chromatin organization” were all enriched within DEGs of the three species. However, a deeper inspection indicated differences in timing and direction of transcriptional changes of genes included into these functional groups. Differences could easily be detected not only between but also within species, with clear divergences among HCR and LCR genotypes at level of both developmental- and environmental related functional categories.

These results highlight that the three species activate similar and conserved pathways of flower development that permit flower organ differentiation during winter, when trees experience low or/and chilling temperatures and accumulate CH. However, the analyses of transcriptomic data confirm that differentiation of the floral bud is an extremely intricate process that needs the coordination of genetic regulators, hormonal and environmental stimuli, and does not involve a single pathway to a main inductive factor but a complex network of interactions between different stimuli.

To gain better insight on species- and genotype-specific transcriptional regulation of flower bud dormancy, we compared the expression profiles of DEGs included in the main functional categories above identified between and within species. Highlighted transcriptional similarities and/or dissimilarities will be discussed in the light of known gene functions and regulative role of gene families.

### MADS-box transcription factors

*MADS-box* genes encode a large family of transcription factors, which control diverse developmental processes in plants. In flowering plants, *MADS-box* gene family members are key regulators of flowering time and floral organ identity. In particular, according to the ABCDE model, petals, sepals, stamens, carpels and ovules are specified by the combinatorial interaction of five different gene classes: sepal identity is determined by class A genes; identity of petals by the combined activity of class A and class B genes; stamens are specified by class B and class C gene activity; carpels by class C genes; and ovule identity is conferred by class D genes. All floral organs and ovules require class E gene function^20^.

**Figure 1:**
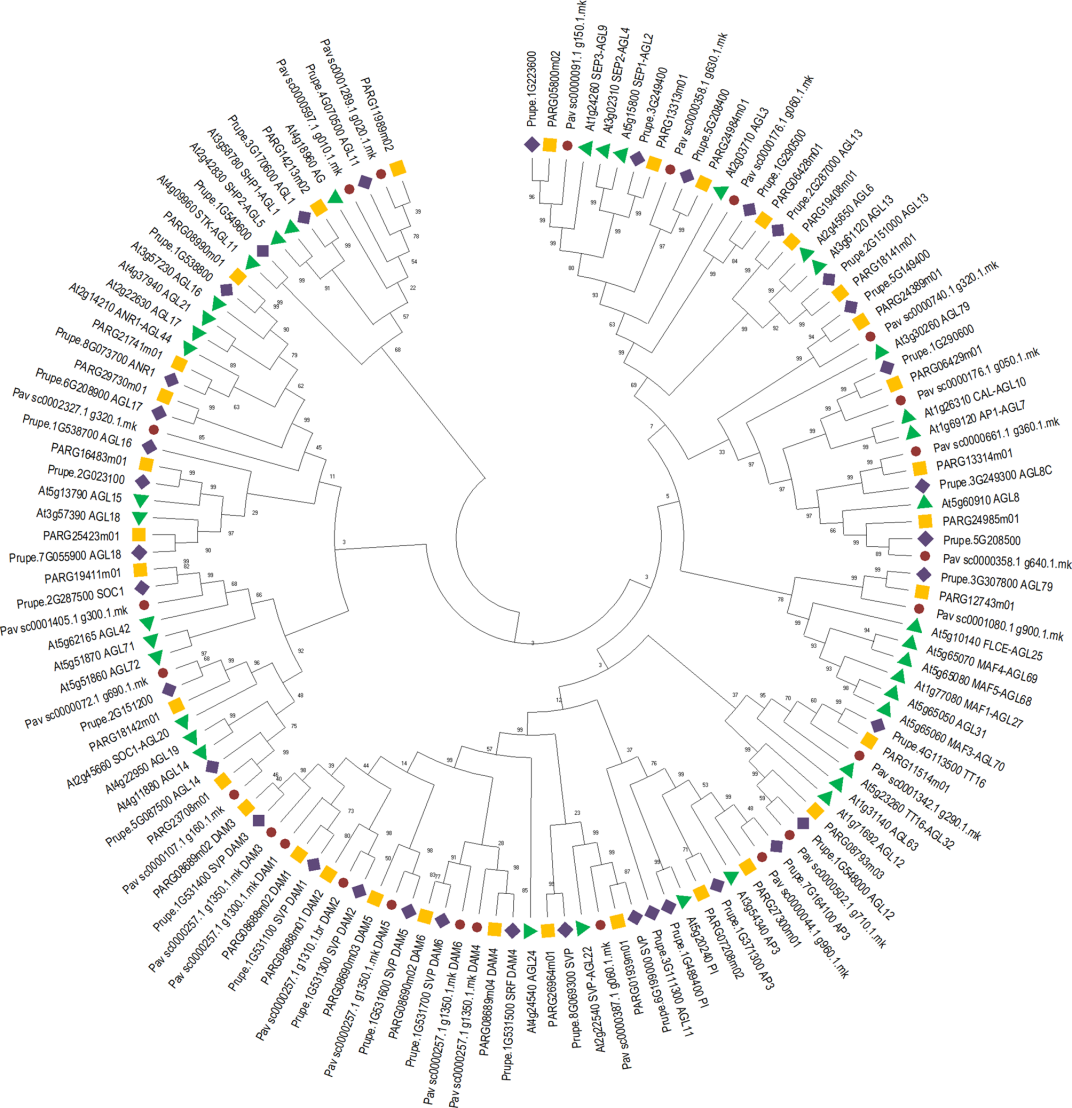
Neighbor-Joining consensus tree of MYKC MADS-box proteins from peach, apricot, cherry and Arabidopsis. The tree is based on a ClustalX alignment and was inferred in MEGAX^58^ with 1000 bootstraps using the Neighbor-Joining method^59^. The evolutionary distances were computed using the JTT matrix-based method^60^ and are in the units of the number of amino acid substitutions per site. The analysis involved 133 amino acid sequences. Green triangles, lilac diamonds, brown circles and yellow squares colors represent Arabidopsis, peach, sweet cherry and apricot proteins respectively.

A phylogenetic analysis (see Supplemental Materials and Methods) was used to separate *Prunus spp* MADS-box proteins into M- and MYKC-type groups (Supplemental Fig. 8) and then to analyze the relationships among genes from the SVP/AGL24 MADS subfamilies, to identify floral homeotic genes involved in the ABCDE functions across multiple species (Fig. 1).

After the identification of MADS subfamily members in each species, a comparative gene expression analysis was performed for a selected number of *MADS* differentially expressed and putatively involved in flower bud dormancy and flower architecture determination. Indeed, our objective was to monitor flower bud dormancy progression in peach, sweet cherry and apricot, through the expression of MADS-box genes.

**Figure 2:**
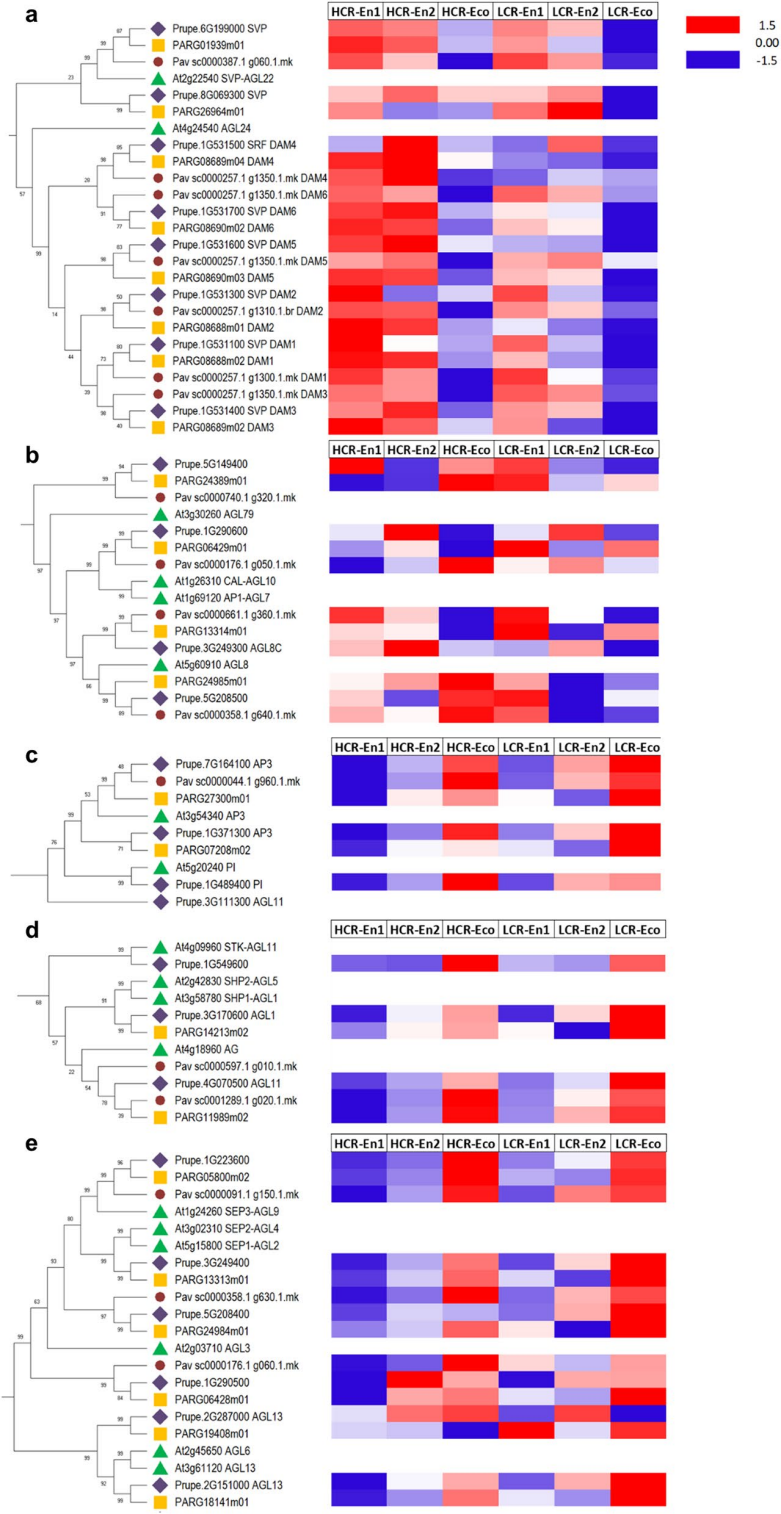
Expression profiles of MYKC MADS-box family genes in flower buds of peach, apricot and sweet cherry during the endodormancy to ecodormancy transition. For *Prunus spp.* genes belonging to AGL24/SVP/DAM (**a**), AP1/CAL (**b**), AP3/PI (**c**), AG (**d**) and SEP (**e**) clades extracted from the tree in Fig. 1, expression profiles in endodormancy entrance (En1), endodormancy release (En2) and ecodormancy (Eco) are reported for HCR and LCR genotypes. Heat maps show the z-score scaled gene expression levels (red indicating higher and blue lower expression, respectively) of genes identified as DEGs in the differential expression tests. Rows corresponding to Arabidopsis genes or to *Prunus spp.* genes not differentially expressed are depicted in grey. Green triangles, lilac diamonds, brown circles and yellow squares colors represent Arabidopsis, peach, sweet cherry and apricot proteins respectively.

*DAM* genes are related to *AGAMOUS-LIKE 24* (*AGL24*) and *SHORT VEGETATIVE PHASE* (*SVP*) gene of Arabidopsis. *SVP* has one and two orthologs in sweet cherry, peach and apricot, respectively. In Rosaceae, both *DAM* and *SVP* transcripts are differentially regulated in bud endodormancy and ecodormancy, and their downregulation is associated to bud burst. In peach, it has been reported that the expression of *DAM* genes is higher in cultivars with a high chilling requirement prior to chilling while it drops down when bud break competence is reached in all cultivar types^21^. In addition, in peach a deletion of a series of *DAM* genes resulted in loss of endodormancy induction^22^. Accordingly, the results of our expression analysis showed that both *DAM* and *SVP* transcripts are strongly downregulated in ecodormancy, both in HCR and LCR genotypes of peach, sweet cherry and apricot. Only for a *PpeSVP* ortholog, ecodormancy transcriptional downregulation is not complete in HCR genotypes. In spite of their sequence homology and based on their expression dynamic, our results indicate that *DAM* gene sub-functionalization has differently occurred in the three studied species. However, they can be considered good markers of the bud endodormancy phase (Fig. 2a).

The *APETALA1* (*AP1*) and *Cauliflower* (*CAL*) are two closely related genes that belong to the A-class homeotic function as well as being meristem identity genes. *AP1* is expressed only in flower whorls 1 and 2, where it is required for sepal and petal development. In Arabidopsis, *AP1* controls the expression of *AGL8*, which RNA accumulates to high levels in the inflorescence apical meristems in the inflorescence stem and cauline leaves and also in the walls of the developing carpels^23^. *Prunus AP1/CAL-like* transcripts are differentially expressed during bud dormancy in peach, apricot and sweet cherry: each species appears to accumulate *AP1* transcripts differently in endo- and eco-dormancy as well as in HCR and LCR genotypes (Fig. 2b). Two *AGL8-like* sequence groups were identified as differentially expressed in the three studied species during bud dormancy: one group collects genes that are preferentially expressed in endodormancy in peach and sweet cherry and apricot HCR, while their expression is really variable in LCR genotypes; the second group members are upregulated in ecodormancy in peach, sweet cherry and apricot HCR genotypes, while their transcripts accumulate in early endodormancy in all LCR genotypes. A similar expression pattern is shared between this *AGL8*-like group and a further group of this MADS subfamily that forms a separate cluster in the phylogenetic tree (Fig. 2b).

*APETALA3* (*AP3*) and *PISTILLATA* (*PI*) family of MADS-box genes encodes a floral homeotic class B-function MADS-box protein and plays crucial roles in petal and stamen development. Two different *AP3* genes are differentially expressed in peach and apricot and one in sweet cherry during bud dormancy. The two identified *AP3* transcripts show an overlapping expression pattern in peach and sweet cherry. In apricot, the same genes are mainly expressed in ecodormancy. Only the *PI* ortholog of peach was found as differentially expressed during flower bud dormancy (Fig. 2c).

*AGAMOUS*- (*AG*-) like genes in Arabidopsis comprise *AG*, *AGL11*, *SHATTERPROOF 1* (*SHP1*), and *SHP2*. Particularly, *AG* was the first class of C floral homeotic gene ever cloned^24^ and it is involved in specifying stamen and carpel identity and in providing floral determinacy. Specifically, *AGL11* controls genes of ovule identity. In peach, sweet cherry and apricot, the orthologs of *AGL11* and *SHP1* are expressed in ecodormancy, both in HCR and LCR (Fig. 2d).

The *SEPALLATA 1*, *2*, *3* (*SEP1, 2, 3*) MADS-box genes are closely related in sequence and in many plant species they share overlapping expression patterns early during flower development. *SEP* genes are thought to define a new class of organ identity function required for the activities of the B- and C-class genes at post-transcriptional level, through protein–protein interactions. These characteristics of *SEP* genes are consistent with the gene expression pattern observed in our analysis, which indicates a substantial upregulation of *SEP* genes in ecodormancy in HCR and LCR genotypes in peach, sweet cherry and apricot (Fig. 2e).

Arabidopsis *AGL13* is a member of the *AGL6* clade of the MADS-box gene family, which belongs to class E and acts as activator of early initiation and further development of pollen and ovules. In peach and apricot, two differentially expressed putative orthologs of *AGL13* were identified and upregulated differently during dormancy (Fig. 2e).

### Hormones

Although the exact mechanisms by which dormancy is regulated by plant hormones is still only partially understood, ABA, GAs and auxins are considered the major hormones that control bud growth cessation, dormancy and activity resumption.

**Figure 3:**
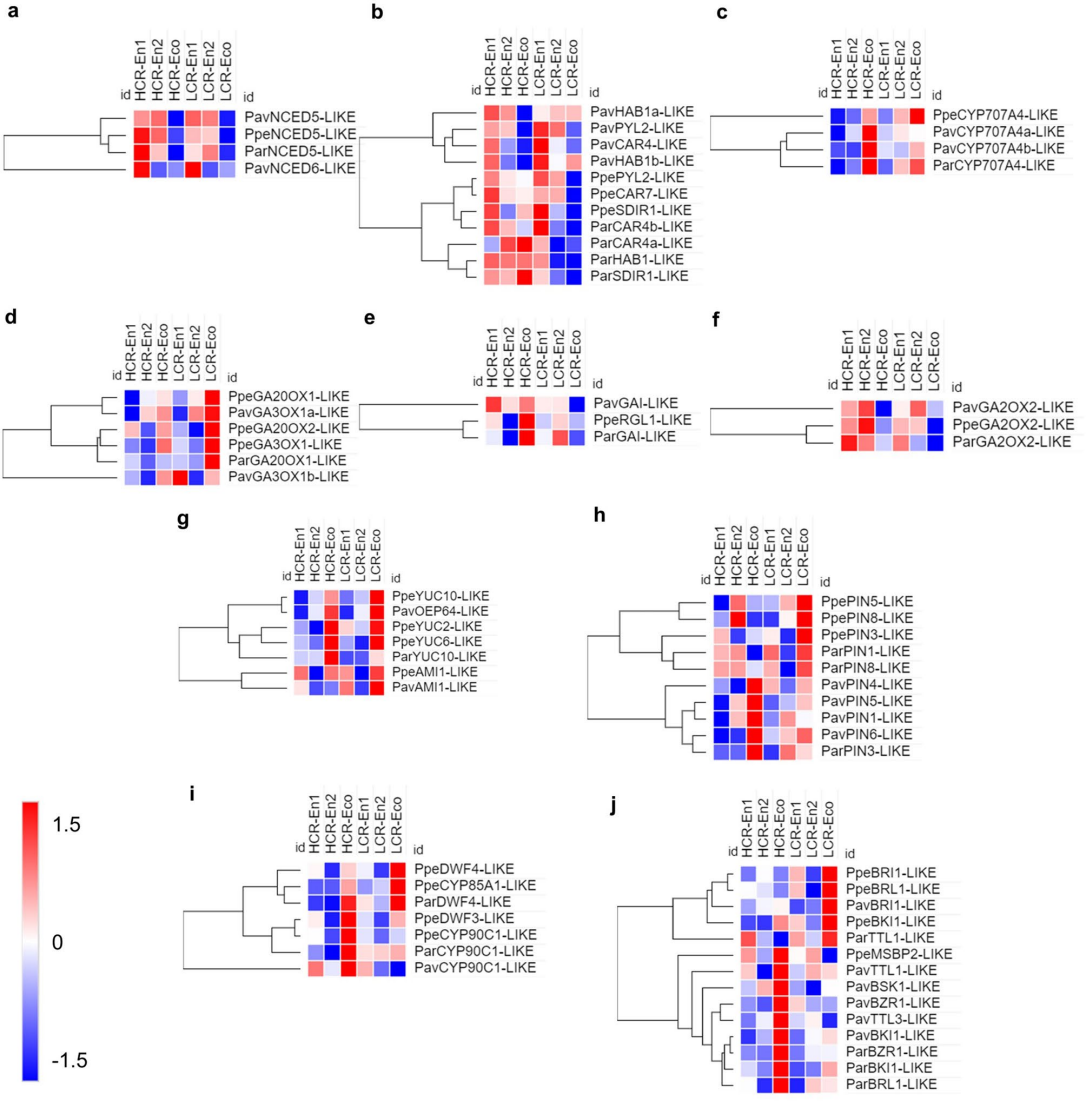
Independent heat maps were prepared for genes involved in ABA synthesis (**a**), signaling (**b**) and catabolism (**c**), GA synthesis (**d**) signaling (**e**) and catabolism (**f**) auxin synthesis (**g**) and transport (**h**), brassinosteroid synthesis (**i**) and signaling (**j**). Expression profiles of differentially expressed genes in endodormancy entrance (En1), endodormancy release (En2) and ecodormancy (Eco) in peach (Ppe), apricot (Par) or sweet cherry (Pav). For each species HCR and LCR genotypes are considered. z-score scaled DEGs expression levels are reported in red to blue color scale, corresponding to higher and lower expression, respectively.

The observed increasing ABA content at dormancy and decreasing at dormancy release in flower buds indicated that it is involved in the maintenance of endodormancy^25^. The DEGs related to ABA synthesis, signaling and catabolism for the three species are reported in Fig. 3. In ABA biosynthesis, *9-cis epoxycarotenoid dioxygenases* (*NCEDs*), which catalyze the critical step for ABA synthesis, are highly transcribed at the beginning of endodormancy in all three species, confirming that ABA is implicated in dormancy entrance (Fig. 3a). In the same way, ABA receptors are upregulated during endodormancy. Pyrabactin resistance (PYR)/Regulatory component of the ABA receptor (RCAR) protein family serve as ABA receptors and when they are bonded to ABA, they inactivate type 2C protein phosphatases (PP2C; Fig. 3b). Conversely, expression of ABA hydroxylase catabolic genes is rapidly increased in ecodormancy, suggesting that these genes are participating in the change/transition of dormancy status (Fig. 3c).

GAs promote plant growth by stimulating plant cell division and/or elongation. GA 20-oxidase (GA20ox) and GA 3-oxidase (GA3ox) are two important dioxygenases involved in conversion of GA to bioactive GAs. Particularly in *Prunus mume*, it was reported that GAs content changes during dormancy, accordingly to the expression of biosynthetic *GA20ox* genes^26^. Both *GA20ox* and *GA3ox* genes are highly expressed towards the end of endodormancy and during ecodormancy, indicating that they may be involved in endodormancy exit (Fig. 3d). GA 2-oxidase (GA2ox) is responsible for GAs deactivation. Its expression is tightly developmentally regulated and upregulated by GAs. In peach, apricot and cherry its transcript level decreased during ecodormancy (Fig. 3f). These observations on GA2ox are consistent with the downregulation observed by Yu et al.^27^ in a recent study on gene expression in peach and apricot bud dormancy. In HCR genotypes at ecodormancy, *GA2ox* expression pattern is opposite to that of DELLA proteins, which act as the negative regulators of GA signaling and inhibitors of plant growth (Fig. 3e). All together these expression data corroborate previous reports which suggested that ABA has a function opposite to that of GA in bud dormancy. They also suggest that an increase in bioactive GA levels after the transition from endodormancy to ecodormancy might be responsible for the acquisition of competence to reactivate bud growth.

The flavin-containing monooxygenase (*YUCCA*) gene family has a key role in auxin biosynthesis and its members are differentially expressed according to plant tissue. These genes encode for the enzymes responsible for oxidizing the intole-3-pyrunvae (IPA) to IAA. *YUCCA* genes are upregulated in both HCR and LCR genotypes during ecodormancy, suggesting that auxin synthesis is active during this phase (Fig. 3g).

In plant PIN efflux carriers are responsible for setting auxin flux associated to tissues and organs differentiation^28^. *PIN* transcripts are differentially regulated during dormancy in the three species analyzed in HCR and LCR genotypes (Fig. 3h). However, a common feature of these gene family members is the upregulation in ecodormancy, in both HCR and LCR genotypes. In summary, the results regarding both auxin synthesis and its polar transport indicate that auxin has a role in the differentiation and resumption of growth processes that occur at ecodormancy.

Brassinosteroids (BR) are steroidal hormones with compounds present in almost all plant organs, with the highest concentrations in pollen grains and immature seeds. The genes encoding for Steroid 22-alpha-hydroxylase (DWF4), steroid 3-dehydrogenase (Constitutive Photomorphogenesis and Dwarfism; CPD) and 3-epi-6-deoxocathasterone 23-monooxygenase (Cytochrome; CYP), the three main enzymes involved in BR biosynthesis, are highly expressed during ecodormancy in peach, apricot and cherry, in particular in HCR cultivars (Fig. 3i). Similarly, the genes encoding for the BR signaling pathway are up regulated in ecodormancy in both HCR and LCR genotypes (Fig. 3j). All together these results indicated BR-related genes as good markers of ecodormancy.

### Megasporogenesis and megagametogenesis

**Figure 4:**
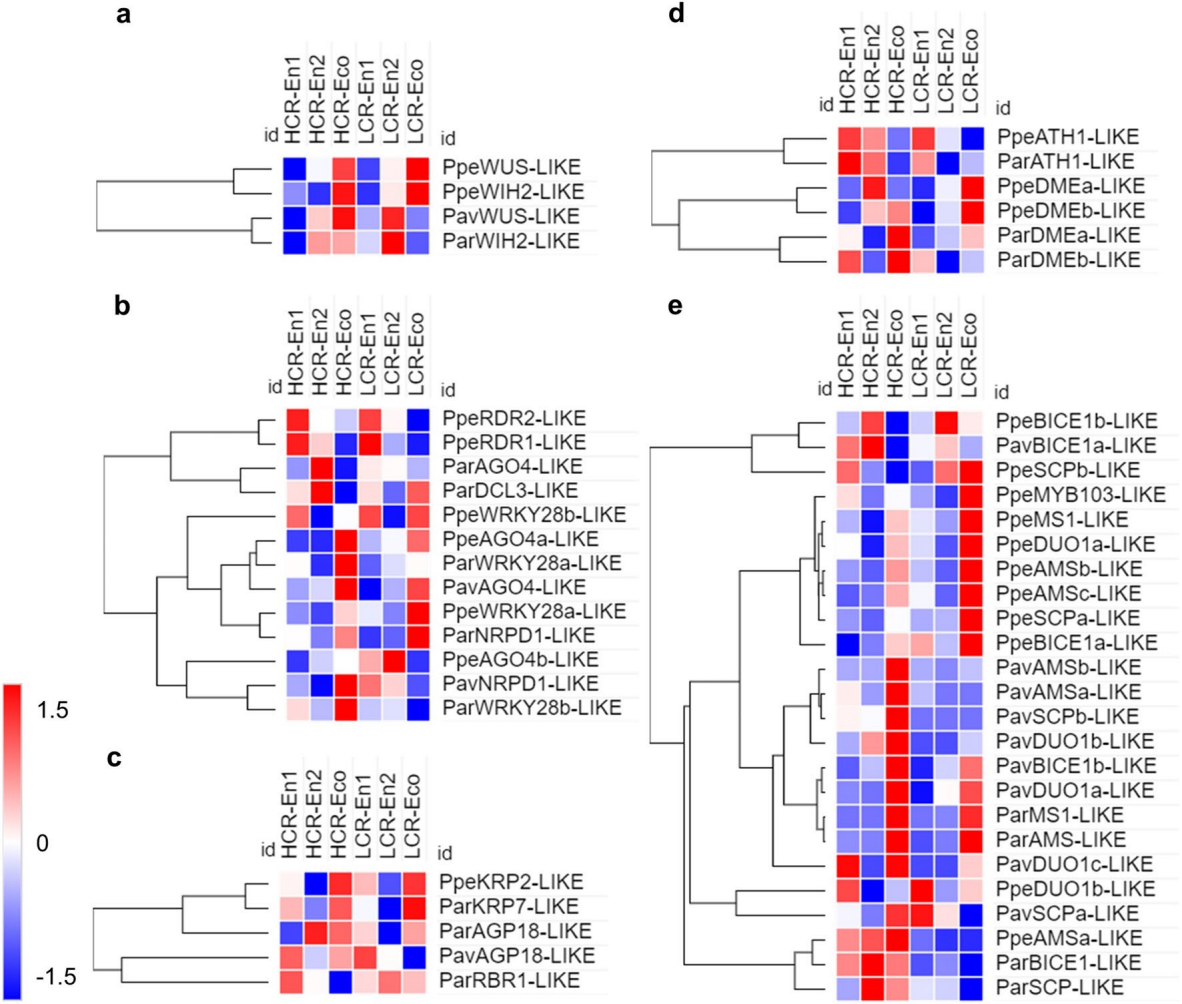
Expression profiles of differentially expressed genes involved in megasporogenesis (**a**–**d**) and microsporogenesis (**e**) during endodormancy entrance (En1), endodormancy release (En2) and ecodormancy (Eco) in peach (Ppe), apricot (Par) or sweet cherry (Pav). For each species HCR and LCR genotypes are considered. z-score scaled DEGs expression levels are reported in red to blue color scale, corresponding to higher and lower expression, respectively.

The female gametophyte, or embryo sac, develops into the ovule located in the gynoecium of the flower. In dicots, such as Rosaceae, the mature embryo sac contains seven cells: centrally positioned egg and central cell flanked by three antipodal and two synergid cells. To determine how and when gametophyte progression might occur during flower bud dormancy, the transcripts of genes known to be involved in gametophyte development in model plants^29^ were analyzed for their differential expression. During early ovule development the megaspore mother cell (MMC) undergoes meiosis to produce four haploid megaspore cells, three of which degenerate, leaving one functional megaspore to generate the embryo sac. Interestingly in peach, both *WUSCHEL-like* (*WUS*) transcription factor and *WINDHOSE2-like* (*WIH2*) gene encoding a small peptide that are known to control MMCs formation in Arabidopsis are differentially expressed during dormancy and upregulated in ecodormancy^29^. In sweet cherry and apricot, only *WIH2* or *WUS*, respectively, were detected as differentially expressed and their expression pattern appeared genotype-dependent: upregulated in both late endodormancy and ecodormancy in HCR genotypes, only in late endodormancy in LCR (Fig. 4a).

It has been proposed that epigenetic regulation is pivotal to spatially restrict the acquisition of germline fate to a single cell around MMCs in the ovule. One of the targets of epigenetic regulation has been to identify transcription factors in *WRKY28*, expression of which suppresses MMC identity in adjacent nucellar cells^29^. Accordingly, in peach, sweet cherry and apricot *WRKY28-like* sequences are preferentially expressed in ecodormancy (Fig. 3b). Furthermore, epigenetic regulation of MMCs identity also involved the RNA-dependent DNA Methylation (RdDM) epigenetic pathways^29^. Expression of the RdDM pathway components (AGO-4 like, NRPD1; DCL3 and RDR1/2) have a less conserved pattern in flower buds of the three species and their transcripts are accumulated in different phases during dormancy, depending both on the species and genotype (Fig. 4b).

In the following stage of megasporogenesis, when meiosis occurs, the expression of genes related to cell cycle regulation, such as the *KIP-RELATED PROTEIN* (*KRP*) and the *RETINOBLASTOMA-RELATED1* (*RBR1*) genes are upregulated. RBR1, a transcriptional repressor known to control cell differentiation^30^, in turn, directly suppresses WUS activity, which allows the MMC to enter meiosis. *KRP-like* genes are upregulated in peach and apricot in particular in ecodormancy (Fig. 4c). In apricot a strong downregulation of *RBR1-like* gene is also observed in ecodormancy in HCR genotypes (Fig. 4c). In Arabidopsis, other genes expressed in FM comprise *AGP18*, which encodes a plasma membrane-attached glycosylated protein, and *ATH1* (*Arabidopsis Thaliana Homeobox 1*), a BEL1-like homeodomain (HD) transcription factor, known to be involved in regulating the expression of the *GA20ox1* gene^29^. In apricot, *AGP18-like* transcript is expressed in ecodormancy, while at the same time in sweet cherry its downregulation is evident in LCR genotypes. ATH1-*like* transcripts are strongly downregulated in ecodormancy, in both peach and sweet cherry.

Finally, the Arabidopsis DEMETER (DME) DNA glycosylase is expressed in the embryo sac central cell and demethylates the maternal genome before fertilization. It is essential for seed viability, by preferentially targeting small transposons flanking coding genes and initiating plant gene imprinting^31^. *DME-like* transcripts are differentially expressed during dormancy in peach and apricot, while no paralog of *DME* was found differentially expressed in sweet cherry. In peach, HCR and LCR genotypes differentially upregulated two *DME-like* transcripts. In apricot, one *DME-like* sequence is upregulated in ecodormancy; a second transcript is downregulated after early endodormancy in LCR genotypes (Fig. 4d).

Taken together these results regarding the differential expression of genes involved in megasporogenesis and megagametogenesis indicate that development and differentiation of the female gametophyte is most probably initiated in late endodormancy and continues during ecodormancy. The different expression profiles of numerous genes between the HCR and LCR genotypes suggests that the timing and expression patterns that regulate progression of the female gametogenesis might be different in the two kinds of varieties. It will therefore be interesting to investigate the role of environmental conditions on the progression of female gametophyte development.

### Microsporogenesis and microgametogenesis

In flower buds, male reproductive development occurs with formation of the stamen and the differentiation of anther tissues. Within the anther, male meiosis produces the microspores, which will develop into pollen grains. In plants, gene expression and functional analysis studies have identified and characterized a large number of genes that are expressed during pollen development^32^. Our meta-analysis aimed at identifying gene transcripts regulating male meiosis in the three studied species and defining the time of their activation during dormancy by analyzing their differential expression. The majority of conserved genes involved in male gametophyte development and expressed during dormancy in peach, apricot and sweet cherry, are genes participating in meiosis and its genetic control (data not shown). All these genes are expressed in the different phases of meiosis: initiation, double strands break, DNA exchange, and cross-over^32^ which mainly coincide with ecodormancy, in both HCR and LCR genotypes of peach and sweet cherry. In apricot, the homologous genes have more complex expression patterns: in HCR genotypes meiotic genes are preferentially expressed in early endodormancy; in LCR the same genes are preferentially expressed in early endodormancy and ecodormancy.

During gametogenesis, three sporophytic genes *MALE STERILE1* (*MS1*), *ABORTED MICROSPORES* (*AMS*) and *MYB103*, which are expressed in the anther tapetum in Arabidopsis at onset of pollen development^32^, are necessary for normal microspore evolution into pollen. The homologs of these three genes are expressed in ecodormancy in both HCR and LCR (with higher level of transcripts) genotypes in peach and apricot. In sweet cherry, these genes are expressed exclusively in HCR genotypes in ecodormancy (Fig. 4e). *DUO POLLEN1* (*DUO1*) is a transcription factor which is restricted to the male germline and is first detected shortly after the asymmetric division that segregates the germ cell lineage^32^. The homologs of *DUO1* are expressed in peach and sweet cherry mainly in ecodormancy. *SIDECAR* (*SCP*) and *BICELLULAR POLLEN1* (*BICE1*) are two Arabidopsis gametophytic genes expressed in pollen and involved in the progression of mitotic division in the male gametophyte^32^. These genes are mainly expressed in ecodormancy in peach and sweet cherry, while in apricot they are expressed only in endodormancy in HCR genotypes.

At transition between endo and ecodormancy, expression data in the three species support anatomical observations of uncompleted gamete formation and indicate that micro- and macrosporogenesis occur at late ecodormancy. On the basis of these pieces of evidence it can be suggested that genes involved in the regulation of fertile verticil development can be usefully adopted as markers of dormancy progression and boundaries between endo and ecodormancy. However, although some pathways appear to be conserved and this allows conserved marker genes both for endo-and ecodormancy to be identified, some important diversifications exist in terms of differentially expressed genes and timing of gene up- or down-regulation. Particularly, LCR and HCR genotypes can either change the time of transcript up- and down-regulation or apparently activate alternative pathways during bud dormancy.

In addition, the overlapping between the expression profile of microgametogenesis genes and those involved in BRs metabolism and signaling during ecodormancy is worthy of note. The regulatory role played by BRs on the expression of genes involved in the development of pollen grains is largely documented^33^; BRs and male related genes can therefore be usefully adopted as markers of ecodormancy stage and the completion of flower development before bud break.

### Epigenetic regulators

**Figure 5:**
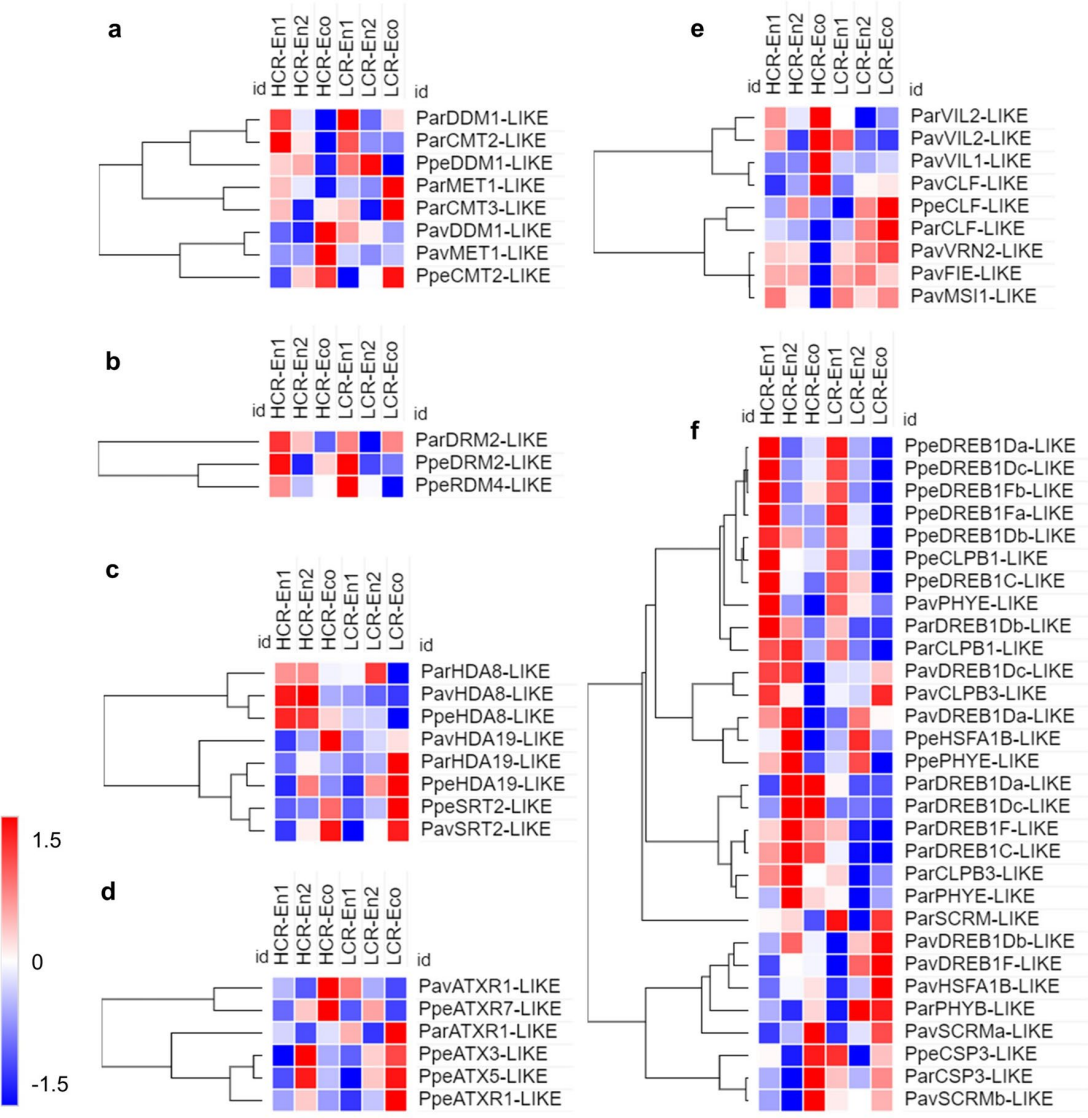
Expression profiles of differentially expressed genes coding for epigenetic regulators: DNA methyltransferases (**a**), gene of RdDM pathway (**b**), histone deacetylases HDA (**c**) and histone methylases of ATX family (**d**) and Polycomb Repressive Complex 2 (PRC2) components (**e**). (**f**) represents the heat-map of genes regulated by light and temperature during endodormancy entrance (En1), endodormancy release (En2) and ecodormancy (Eco) in peach (Ppe), apricot (Par) or sweet cherry (Pav). For each species HCR and LCR genotypes are considered. z-score scaled DEGs expression levels are reported in red to blue color scale, corresponding to higher and lower expression, respectively.

Epigenetic marks include DNA methylation and an array of histone post-translational modifications (PTMs), such as acetylation, methylation, phosphorylation and sumoylation, which play critical roles in regulating DNA replication, DNA repair and RNA transcription^34^. In plants, DNA methylation plays a role in gene regulation and transposon silencing and the Decrease in DNA Methylation 1 (DDM1) of Arabidopsis thaliana is a nucleosome remodeler that enables cytosine methylation of nucleosome-wrapped DNA^35^. *DDM1-like* genes are differentially expressed in peach, apricot and sweet cherry during flower bud dormancy. In peach, *DDM1-like* transcripts accumulate in endodormancy independently of the genotype; similarly, in apricot, *DDM1-like* expression is upregulated early in endodormancy, while in sweet cherry the transcripts accumulate mainly in early endodormancy in LCR genotypes and ecodormancy in HCR genotypes (Fig. 4a). METHYLTRANSFERASE 1 (MET1), the plant homolog of mammalian DNMT1, is responsible for maintaining methylation at CG sites^36^. A *MET1-like* gene is differentially expressed in apricot and sweet cherry (Fig. 4a). *CHROMOMETHYLASE 3* (*CMT3*) is a plant specific gene, the product of which maintains DNA methylation in CHG sequence contexts^35^. *CMT3-like* gene was detected differentially expressed exclusively in apricot in early endodormancy and in ecodormancy (Fig. 5a). A third plant gene *CHROMOMETHYLASE 2* (*CMT2*) maintains CHH methylation at heterochromatic regions, independently of RdDM pathway and small RNAs production^35^. *CMT2-like* genes are expressed in peach in HCR genotypes in late endodormancy and ecodormancy and in apricot in early endodormancy (Fig. 5a). Finally, the plant specific RdDM pathway involves the DOMAINS REARRANGED METHYLASES 1 and 2 (DRM1/2) for maintaining methylation in all sequence contexts^35^. A *DRM2-like* gene is differentially expressed in peach and apricot (Fig. 5b). Interestingly, in peach another gene involved in the RdDM pathway, *RDM4-like* gene is highly expressed at the beginning of endodormancy (Fig. 5b). *RDM4* encodes a protein that is required for RNA-directed DNA methylation in Arabidopsis, with a role in cold stress response.

The expression of the genes responsible for the maintenance of DNA methylation in the three studied species highlights important differences in the timing of gene transcript accumulation, suggesting that DNA methylation might be used differently to regulate the gene cis-regulatory sequence accessibility to the transcriptional machinery, during dormancy in the *Prunus* species analyzed. To support this view, a global analysis of methylation status on flower buds of almond, another species belong to the *Prunus* genus, pointed out that more hypermethylated than hypomethylated fragments were identified in dormant buds independently of genotype^37^.

In plants, histone acetylation/deacetylation studies characterized a numerous *HDA* gene family, the members of which remove acetyl groups that are deposited by histone acetyltransferases (HATs) in histones^38^. During bud dormancy, we observed that some members of the *HDA* family are specifically expressed in peach, apricot and sweet cherry (Fig. 5c). In particular *HDA19-like* gene is expressed in late endodormancy in peach HCR genotypes and in ecodormancy in LCR; similarly, the gene is expressed in ecodormancy in all sweet cherry genotypes and in LCR genotype of apricot. Among the many regulatory roles, AtHDA19 was shown to participate in flower organ differentiation, in ABA and BR signaling^38^. Another HDA family member *HDA8-like*, which has not yet been functionally characterized in plants, is also differentially expressed in all three species during dormancy. For all species, *HDA8-like* is mainly expressed during endodormancy in HCR, while in LCR genotypes it is downregulated in ecodormancy (Fig. 5c). Sirtuin-like HDAs require NAD + as a cofactor for their enzymatic activity; only one or two sirtuin-like (SRT) proteins are commonly present in plants^38^. In peach and sweet cherry *SRT2-like* gene is expressed in ecodormancy (Fig. 5c). Other HDAs members are specifically expressed in one or two out of the three species, thus indicating that the mechanisms through which they regulate chromatin activities might be species dependent.

Histone methylation/demethylation contributes to chromatin dynamics and gene transcription regulation in plants. Particularly, dynamic reprogramming of two antagonistic marks, histone H3 lysine tri-methylation (H3K4me3) and histone H3 lysine 27 tri-methylation (H3K27me3), occurs in many regulatory genes. H3K4 methylation is mainly mediated by the Trithorax (TrxG) group proteins ATX and ATX related proteins (ATXR). Our meta-analysis results showed that *ATXR1-like* genes are differentially expressed during dormancy, although with a different pattern of expression in the studied species (Fig. 5d). In peach, two other members of the ATX and ATXR families are upregulated in late endodormancy in HCR genotypes and in ecodormancy in LCR genotypes (Fig. 5d). Deposition of the antagonistic H3K27me3 mark is mediated by Polycomb Repressive Complex 2 (PRC2). In plants, four conserved subunits with alternative component combinations form diverse PRC2 complexes, playing crucial roles in distinct plant developmental phases. We observed that in sweet cherry four *PRC2* components share the same expression pattern: *CLF-like*, *VRN2-like*, *FIE-like* and *MSI1* are expressed in endodormancy and ecodormancy of LCR but are strongly downregulated in ecodormancy of HCR genotypes (Fig. 5e). Interestingly, *VIN3-like protein 1* and *2* (*VIL1* and *VIL2*) genes were identified as differentially expressed genes during dormancy in apricot and sweet cherry (Fig. 5e). In Arabidopsis, these proteins belong to a small family of PHD finger proteins, that during the prolonged cold are necessary for establishing the vernalization-induced chromatin modifications.

The identification of some epigentic regulators and the dynamics of their expression indicate that chromatin states can be modified during bud dormancy by epigenetic marks in the three species under investigation. In addition, these data confirm that differences are evident between species and HCR and LCR genotypes in modulating the epigenetic regulator expression and mechanisms controlling chromatin changes.

### Temperature and light

One major function of dormancy is the protection of developing flower tissues inside the buds from cold. The prolonged exposure to low winter temperatures that overlaps with endodormancy, guarantees the fulfillment of a CR, which is critical to ensure an optimal flowering in spring and is considered to be specific for each variety or genotype. We thus investigated the expression pattern of cold-response genes among those transcripts that were identified as DEGs during the endodormancy to ecodormancy transition to evaluate whether cold acclimation responses provoke similar changes in gene expression (Fig. 5f).

In both peach and apricot, *CBF/DREB1D* (C-repeat-binding factor/dehydration responsive element-binding factor 1D) transcripts are upregulated in the initial phase of endodormancy and also in late endodormancy but only in HCR genotypes. In peach, there are two additional *CBF/DREB1D* transcript sequences behaving like specific gene markers of early endodormancy. Additional apricot *CBF/DREB1D* transcripts are upregulated in late endodormancy and ecodormancy exclusively in HCR genotypes. Instead, *CBF/DREB1D* transcripts in sweet cherry are upregulated in endodormancy or in ecodormancy, mainly depending on the genotype. Other members of the CBF/DREB1 family, namely CBF/DREB1F and CBF/DREB1C are upregulated during flower bud dormancy in a more specific way (Fig. 5f). In plants, CBF/DREB1 genes encode a small sub family of the AP2/ERF transcriptional activators that plays an important role in freezing tolerance and cold acclimation^39^. In apple, overexpression of peach CBF transcripts delayed bud break and induced cold inducible genes (COR) expression^40^. CBFs bind upstream of DAM genes and induce their expression at early time points^41^.

In sweet cherry, two transcripts of *ICE1* also known as *SCREAM* (*SCRM1*,^42^) mark ecodormancy. One ortholog of *SCRM* is upregulated in early endodormancy and in ecodormancy in apricot LCR genotype. *INDUCER OF CBF EXPRESSION 1* (*ICE1*) encodes a MYC-like basic helix-loop-helix (bHLH) transcription factor regulating *CBF/DREB1D* and playing multiple roles in Arabidopsis, from responses to chilling and freezing stresses to leaf stomata development, male fertility control and water movement in the anthers^43^.

Peach and apricot share the expression patterns of *Casein lytic proteinase B1* (*CLPB1*) transcripts that are upregulated in early endodormancy. In apricot, a second transcript coding for a *CLBP3* protein is specifically expressed during endodormancy. In sweet cherry, the ortholog gene transcript is upregulated in endodormancy in HCR genotypes and in ecodormancy in LCR genotypes. In plants, the cytosolic CLPB1 is a heat shock protein required for acclimation to high temperature. However, the function of ClpB/Hsp100 proteins is not restricted to heat stress: it has been shown that a specific member of the family provides housekeeping functions that are essential to chloroplast development^44^. Thus, it would be interesting to elucidate the role of these proteins, which mark a specific phase of bud dormancy in *Prunus* spp.

*Heat stress transcription factor A-1b* (*HSFA1b*) transcript is upregulated in peach flower bud in late endodormancy; differently, the transcript of sweet cherry ortholog accumulates mainly in ecodormancy and in LCR. In spite of its name, HSFA1b has been proposed to act as a transducer of environmental cues to many stress tolerance and developmental genes to allow plants to continually adjust their growth and development in a varying environment^45^.

In peach and apricot, *CSP3* gene transcripts are upregulated during ecodormancy. Cold shock proteins (CSPs) have essential roles in cold adaptation as RNA chaperones. In particular, Arabidopsis cold shock domain protein 3 (AtCSP3) is involved in the acquisition of freezing tolerance in plants^46^.

Two phytochrome gene transcripts (*PHYB* and *PHYE*) were differentially expressed during dormancy, although *PHYB* is upregulated only in sweet cherry (Fig. 5f). It is well known that through phytochromes plants obtain information about their immediate environment and the changing seasons. In particular, *PHYE* has been demonstrated to play a greater role than *PHYB* in the control of flowering under cooler conditions^47^. This observation appears consistent with the expression during dormancy of *PHYE* in the three studied species.

Interestingly, our meta-analysis provides starting information for detailed investigations on the main pathways involved in bud formation processes and their interactions with endogenous and environmental cues. In addition, identification of transcript variants specifically activated in one genotype can provide information on the genetic variability to canonical responses that might account for different adaptation strategies to the environment. An understanding of all the variables influencing the formation of buds, from flower induction initiation to gamete formation is essential to support breeding programs and cultivation management in the current climate change scenario.

## Materials and methods

### Search of RNA-Seq studies and data set collection for meta-analysis

Transcriptomic studies publicly available before December 2019 in *Prunus spp* were identified in the NCBI Sequence Read Archive (SRA; https://www.ncbi.nlm.nih.gov/sra) and Gene Expression Omnibus (GEO; https://www.ncbi.nlm.nih.gov/geo/) databases using the combination of keywords “RNA-Seq”, “bud”, “dormancy”, “transcriptomics” and “Prunus”. We found two large and multi-species studies that satisfy the above criteria. The first study (PRJNA567655;^27^) included peach (*Prunus persica*; Ppe) and apricot (*Prunus armeniaca*, Par) transcriptome analysis during bud endodormancy to ecodormancy transition. The same biological aspects were investigated in cherry (*Prunus avium*, Pav) in a second study (SRP194120;^19^). For each species, the identified studies encompassed at least three different cultivars with diverse chilling requirements (low CR, LCR; and high CR, HCR), for which flower bud transcriptome was analyzed starting from autumnal dormancy entrance to spring release (Supplemental Fig. 1). A detailed list of samples available for each species and respective metadata (paper information, cultivar and chilling requirement, collection date) is provided in Supplemental Tables 1, 2 and 3.

### Reads mapping and counting

Raw sequence data for all accessions were accessed from NCBI Sequence Read Archive using the SRA Toolkit (https://github.com/ncbi/sra-tools) and the NCBI-NGS toolkit (https://github.com/ncbi/ngs) and mapped using HISAT2 v2.1.0^48^. Unlikely when the original research papers were prepared, a reference genome is now available for each analyzed species. Reference genome assemblies and gene annotations for apricot (v1.0;^49^) and cherry (v1.0.a1;^50^) were downloaded from GDR database (https://www.rosaceae.org/;^51^), while Peach v2.0 genome and gene annotations^52^ were retrieved from EnsemblPlants database (https://plants.ensembl.org/Prunus_persica/Info/Index).

HISAT2 mapping parameters were adjusted to species-specific sequencing read length (paired-end 150 nt for peach, 50nt single end for apricot and 75nt paired-end bp for cherry) and quality control outcomes. Before alignment, Trimmomatic^53^ was used for the trimming of five bases at the 5’ end and of low quality bases at the 3’ end of each apricot and cherry read (HEADCROP:5; TRAILING:5 parameters), and for removal of adapters that were found on right-ends of peach reads (ILLUMINACLIP:TruSeq2-PE:2:30:10 parameter). For mapping, maximum intron size was set to 10,000 while the minimum was 10.

Mapped reads were counted using the feature Counts Subread package v 2.0.1^54^. Gene-level read counts were generated independently for each species, and were based only on uniquely mapped reads.

For the gene-count results, the DAM regions in apricot (chromosome LG2), cherry (chromosome PAV_r1.0chr1) and peach (chromosome Pp01) annotation files were manually corrected using locus annotations from in *Prunus persica* v1.0 assembly (genes ID ppa018667m, ppb017585m, ppa010758m, ppa011123m, ppa010822m, and ppa010714m) and the following Genebank sequence accessions: *P. persica* PpDAM1 (DQ863253), PpDAM2 (DQ863255), PpDAM3 (DQ863256), PpDAM4 (DQ863250), PpDAM5 (DQ863251) and PpDAM5 (DQ863252); *P. avium* PavDAM1(LC544139), PavDAM2 (LC544140), PavDAM3 (LC544141), PavDAM4 (LC544142), PavDAM5 (LC544143) and PavDAM6 (LC544144).

### Principle component analysis (PCA) and hierarchical clustering

For each analyzed species, the structure of the data (i.e. sample dissimilarities between genotypes and sampling dates (chilling hours accumulation) were revealed by sample distance matrix and Multidimensional scaling (MDS) using the R (https://www.r-project.org; v3.6.1) package DESeq2 (v1.30;^55^). Counts-based MDS plots and sample distance matrix allowed the structure of data to be assessed, and to sub-select samples corresponding to different dormancy stages: endodormancy entrance (En1), endodormancy progression (En2) and ecodormancy (Eco) and different chilling requirements genotypes (LCR and HCR; Supplemental Figs. 2, 3 and 4). Based on the data inspections, only two *Prunus armeniaca* genotypes were considered for further analyses. Species-specific gene level abundances were calculated as FKPM (Fragments Per Kilobase Million mapped reads) or RPKM for apricot single-end sequencing.

### Differential expression analysis

Differential expression analyses over time and between HCR and LCR cultivars were performed independently in each species using DESeq2 (v1.30;^55^) Likelihood ratio test (LRT). Differently to the default Wald test, LRT is used to identify any gene that shows a change in its expression across the different levels, resulting particularly useful in analyzing time course experiments. Genes showing a false discovery rate (FDR) below 0.01 were considered as significantly differentially expressed genes (DEGs) during endodormancy release in HCR or in LCR genotypes. Even if in the LRT approach, log2 fold changes are not directly associated with the actual hypothesis test and thus not usually used for DEGs determination, only genes showing at least a twofold change among the analyzed samples were selected for DEGs filtering. A third differential expression analysis between HCR and LCR genotypes was performed to identify the different transcriptional regulation associated to dormancy release in the two genotypes (Genotype X Dormancy interaction test); in this case a more stringent FDR cut-off of 0.001 was applied. Differentially expressed genes identified in each RNA-Seq experiment are listed in Supplemental Datasets 1, 2 and 3.

### Gene enrichment analysis and functional analysis

Functional annotation to *Prunus spp* proteins was assigned using Mercator 4 v.2;^56^) that, producing the description of the function of the protein, assigns the proteins in hierarchical functional categories (called BINs) suitable for Mapman4 functional enrichment studies. DEGs functional enrichment was performed with MapMan^57^ using a Wilcoxon test; resulting p-values were adjusted according to Benjamini and Hochberg. The genes belonging to transcription factors, hormone regulation micro- and macro-sporogenesis and epigenetic regulator bin categories of the three species were extracted from Mercator annotations.

### Heat map construction

The heat maps summarizing and comparing expression levels of DEGs (as reveled by DESeq2 analyses) were constructed using MORPHEUS software (https://software.broadinstitute.org/morpheus/). We used three main time points: endodormancy entrance (En1), endodormancy release (En2) and in ecodormancy (Eco) and the expression values independently calculated in three different *Prunus* species, peach (Ppe), apricot (Par) and sweet cherry (Pav). For each one, cultivars with high and low chilling requirement (CR) were compared. Z-score re-scaled gene expression values are reported on a blue to red scale.

## Supplementary Information

Supplementary Information 1.

Supplementary Information 2.

Supplementary Information 3.

Supplementary Information 4.

Supplementary Information 5.

Supplementary Information 6.

Supplementary Information 7.

Supplementary Information 8.
