# Supplementary material for: Meta-analysis of RNA-Seq studies reveals genes with dominant functions during flower bud endo- to eco-dormancy transition in Prunus species
Source: Sci Rep. 2021 Jun 23;11:13173. doi: 10.1038/s41598-021-92600-6 (PMC8222350; doi:10.1038/s41598-021-92600-6)
Supplement: Supplementary file 8 — Supplementary Information 8. [file 41598_2021_92600_MOESM8_ESM.docx]

**Monica Canton^*^, Cristian Forestan ^†^, Claudio Bonghi^*^ and Serena Varotto^*^**

**^*^Department of Agronomy Food Natural Resources, Animals and Environment (DAFNAE) Agripolis, University of Padova, 35020 Legnaro (PD), Italy, ^†^ Department of Agricultural and Food Sciences (DISTAL) University of Bologna, 40127 Bologna (BO), Italy.**

**Meta-analysis of RNA-Seq studies reveals genes with dominant functions during flower bud endo- to eco-dormancy transition in *Prunus* species**

**SUPPLEMENTARY METHODS**

**MADS-box genes identification and phylogenetic analysis**

Arabidopsis M- and MYKC-type (also known as Type I and II, respectively) MADS coding genes accession, annotation and names were obtained from the TAIR Gene family database (<https://www.arabidopsis.org/browse/genefamily/index.jsp>) and confirmed through the Plant Transcription Factor Database (PlantTFDB; <http://planttfdb.cbi.pku.edu.cn/index.php>). This database was also used for retrieval of *Prunus persica* MADS accessions and annotations. In parallel *P. persica*, *P. avium* and *P. armeniaca* MADS coding genes were identified from the species-specific transcriptome functional annotation obtained from Mercator4. The 81 peach MADS genes retrieved from PlantTFDB were fully confirmed by our Mercator4 annotation, which also identified 3 additional MADS transcription factors, indicating the robustness of this functional annotation tool.

An initial phylogenetic analysis was performed to separate the full set of 327 MADS-box proteins (Supplemental File 1 and Supplemental Figure 7) into M- and MYKC-type using Arabidopsis and peach grouping as scaffold. MADS amino acid sequences were aligned with ClustalX ^1^ and used to create a Neighbor-joining phylogeny tree in MEGAX with 100 bootstraps ^2^. Both ClustalX guide tree and MEGA NJ tree unambiguously separated annotated Arabidopsis and peach M- and MYKC-type proteins into two separated groups. Apricot and cherry proteins were classified based on the branch they belong to. In addition, four subgroups were clearly distinguishable among M-type proteins; based on Arabidopsis annotation they correspond to α, β, γ, and δ clades.

In a second step, protein sequences of MIKC genes from Arabidopsis, peach, cherry and apricot were aligned with ClustalX, and a phylogenetic analysis was performed with MEGAX with 1000 bootstraps using JTT matrix-based method ^3^. Tree was used to analyze the relationships among genes from the SVP/AGL24 MADS subfamilies and to identify floral homeotic genes involved in the ABC functions across multiple species.

**REFERENCES**

1. Larkin, M. A. *et al.* Clustal W and Clustal X version 2.0. *Bioinformatics* **23**, 2947–2948 (2007).

2. Kumar, S., Stecher, G., Li, M., Knyaz, C. & Tamura, K. MEGA X: Molecular evolutionary genetics analysis across computing platforms. *Mol. Biol. Evol.* **35**, 1547–1549 (2018).

3. Jones, D. T., Taylor, W. R. & Thornton, J. M. The rapid generation of mutation data matrices from protein sequences. *Bioinformatics* **8**, 275–282 (1992).

**SUPPLEMENTARY FIGURE 1-8**

**
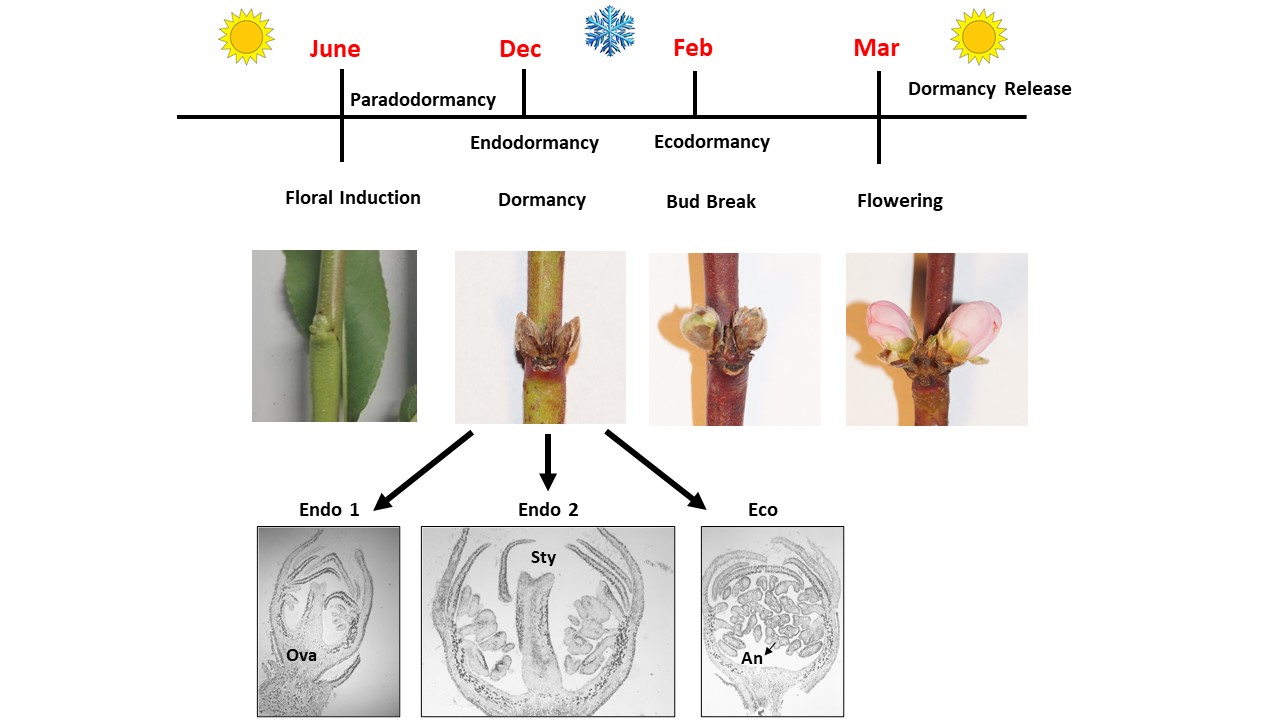
**

**Supplemental Figure 1**

Morphology of the dormancy transition in Peach flower buds starting from floral induction until flowering. Grey images represent longitudinal sections of a peach floral bud during dormancy progression from endodormancy 1 (Endo 1) until ecodormancy (Eco). Ova, Ovary; Sty, Style, An, Anther.


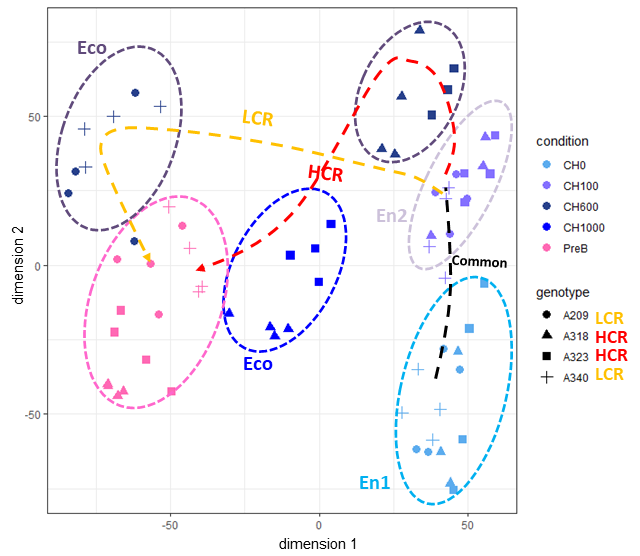


**Supplemental Figure 2**

Multidimensional scaling (MDS) plot of RNA expression profiles in *Prunus persica* (Ppe, peach) flower buds sampled from autumnal dormancy entrance (CH0) to spring release (PreB) from four different cultivars with different chilling requirements (retrieved from Yu et al., 2020). This MDS plot shows that samples cluster according to sampling time-point and genotype chilling requirement, as expected. Samples corresponding to different dormancy stages: endodormancy entrance (En1), endodormancy progression (En2) and ecodormancy (Eco) and different chilling requirements genotypes were therefore sub-selected for meta-analysis study. Genotypes and samples names reported in the legend correspond to those used in the original study (see also Supplemental Table 1). LCR: low chill requirement genotype; HCR: high chill requirement genotype; CH: chilling hours; PreB: pre-blooming stage.


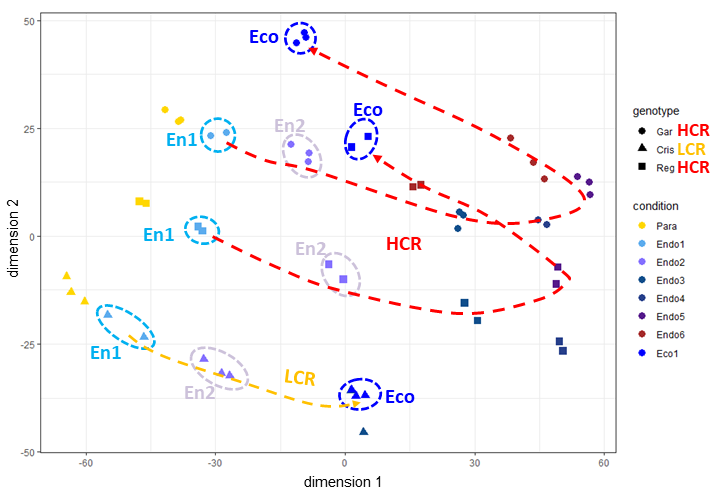


**Supplemental Figure 3**

Multidimensional scaling (MDS) plot of RNA expression profiles in *Prunus avium* (Pav, cherry) flower buds sampled during summer paradormancy (Para), at different points during endodormancy (Endo) and at ecodormancy (Eco), from three different cultivars with different chilling requirements (retrieved from Vimont et al., 2019). This MDS plot shows that samples cluster according to sampling time-point and genotype chilling requirement, as expected. Samples corresponding to different dormancy stages: endodormancy entrance (En1), endodormancy progression (En2) and ecodormancy (Eco) and different chilling requirements genotypes were therefore sub-selected for meta-analysis study. Genotypes and samples names reported in the legend correspond to those used in the original study (see also Supplemental Table 2). LCR: low chill requirement genotype; HCR: high chill requirement genotype.


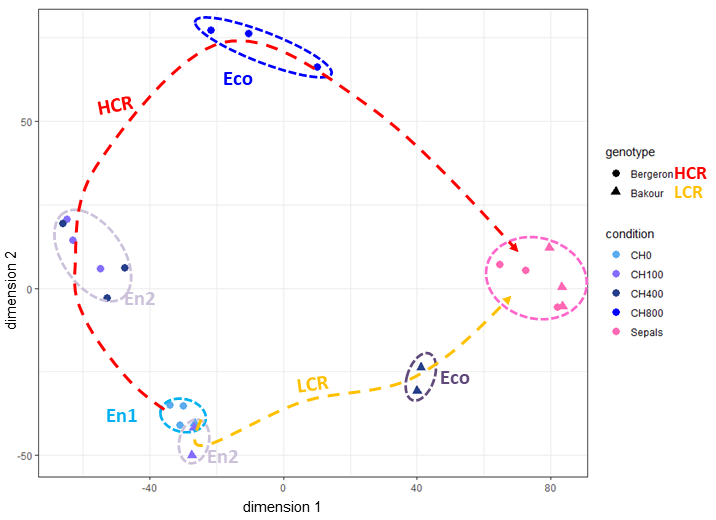


**Supplemental Figure 4**

Multidimensional scaling (MDS) plot of RNA expression profiles in *Prunus armeniaca* (Par, apricot) flower buds sampled from autumnal dormancy entrance (CH0) to spring release (sepals visible) from two different cultivars with different chilling requirements (retrieved from Yu et al., 2020). This MDS plot shows that samples cluster according to sampling time-point and genotype chilling requirement, as expected. Samples corresponding to different dormancy stages: endodormancy entrance (En1), endodormancy progression (En2) and ecodormancy (Eco) and different chilling requirements genotypes were therefore sub-selected for meta-analysis study. Genotypes and samples names reported in the legend correspond to those used in the original study (see also Supplemental Table 3). LCR: low chill requirement genotype; HCR: high chill requirement genotype; CH: chilling hours.


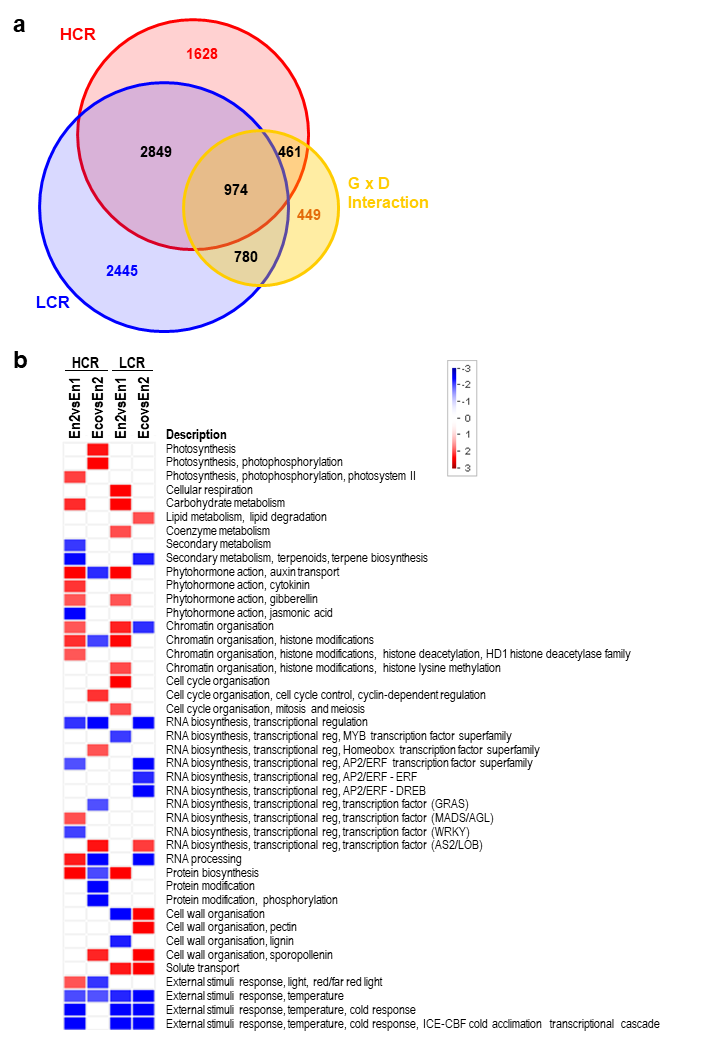


**Supplemental Figure 5**

Results of differential expression analyses in *Prunus persica* (Ppe, peach) flower bud progression from endodormancy to ecodormancy (Eco) in genotypes with different chilling requirements. **A**) Venn diagram summarizing the number of differentially expressed genes (DEGs) identified in each LRT test (HCR genotype, LCR genotype and Genotype x Dormancy interaction (see also Supplemental Dataset 1). **B**) PageMan display of MapMan gene categories regulated during dormancy release in peach. A Wilcoxon rank sum test was used to identify functional groups among DEGs identified in HCR and LCR genotypes using gene expression ratio data for pair-wise sample comparisons. Heat-map indicate statistically significant groups (Benjamini & Hochberg-corrected p-value below 0.05), where the color scale represents z-transformed P-values, with red indicating gene up-regulation and blue, down-regulation.


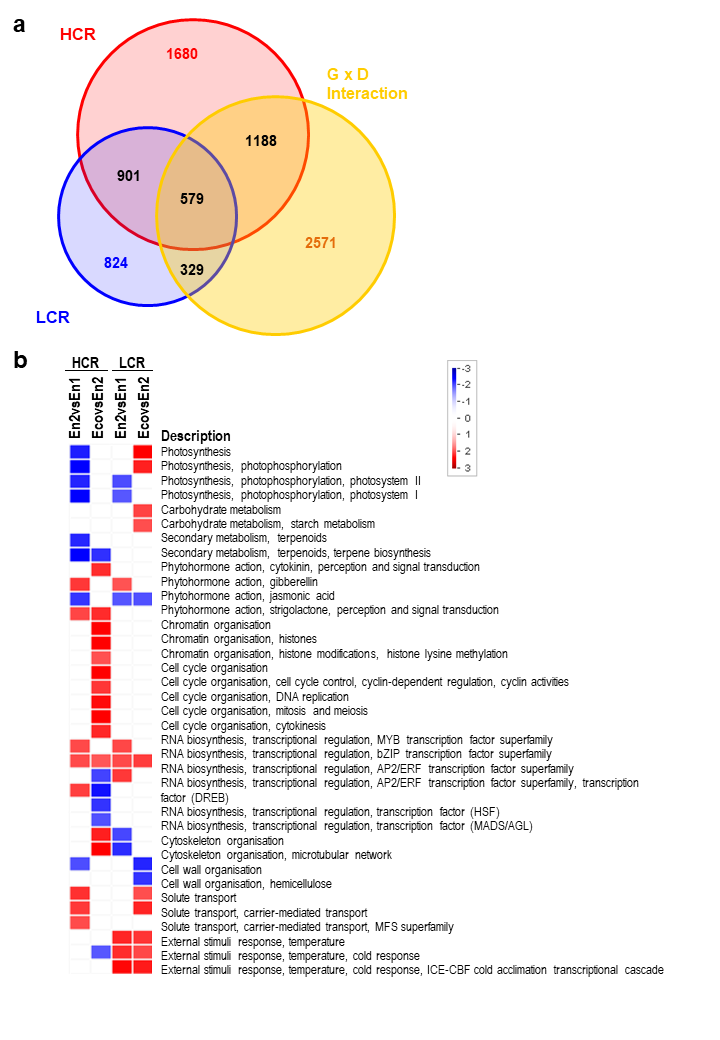


**Supplemental Figure 6**

Results of differential expression analyses in *Prunus avium* (Pav, cherry) flower bud progression from endodormancy to ecodormancy (Eco) in genotypes with different chilling requirements. **A**) Venn diagram summarizing the number of differentially expressed genes (DEGs) identified in each LRT test (HCR genotype, LCR genotype and Genotype x Dormancy interaction (see also Supplemental Dataset 2). **B**) PageMan display of MapMan gene categories regulated during dormancy release in peach. A Wilcoxon rank sum test was used to identify functional groups among DEGs identified in HCR and LCR genotypes using gene expression ratio data for pair-wise sample comparisons. Heat-map indicate statistically significant groups (Benjamini & Hochberg-corrected p-value below 0.05), where the color scale represents z-transformed P-values, with red indicating gene up-regulation and blue, down-regulation.


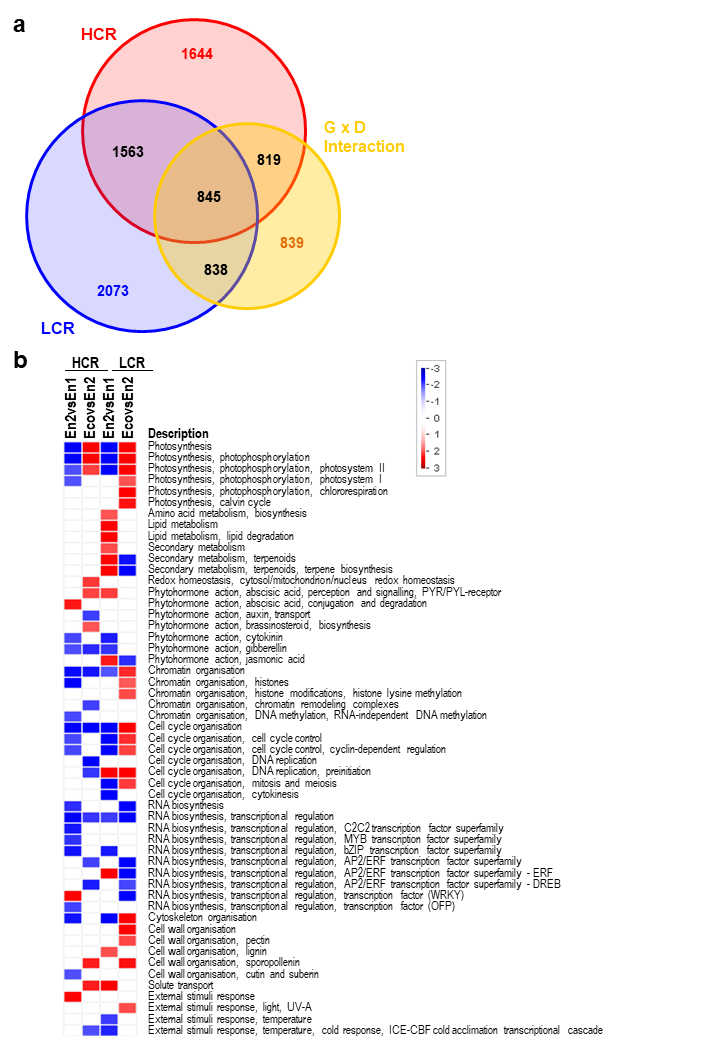


**Supplemental Figure 7**

Results of differential expression analyses in *Prunus armeniaca* (Par, apricot) flower buds progression from endodormancy to ecodormancy (Eco) in genotypes with different chilling requirements. **A**) Venn diagram summarizing the number of differentially expressed genes (DEGs) identified in each LRT test (HCR genotype, LCR genotype and Genotype x Dormancy interaction (see also Supplemental Dataset 3). **B**) PageMan display of MapMan gene categories regulated during dormancy release in peach. A Wilcoxon rank sum test was used to identify functional groups among DEGs identified in HCR and LCR genotypes using gene expression ratio data for pair-wise sample comparisons. Heat-map indicate statistically significant groups (Benjamini & Hochberg-corrected p-value below 0.05), where the color scale represents z-transformed P-values, with red indicating gene up-regulation and blue, down-regulation.


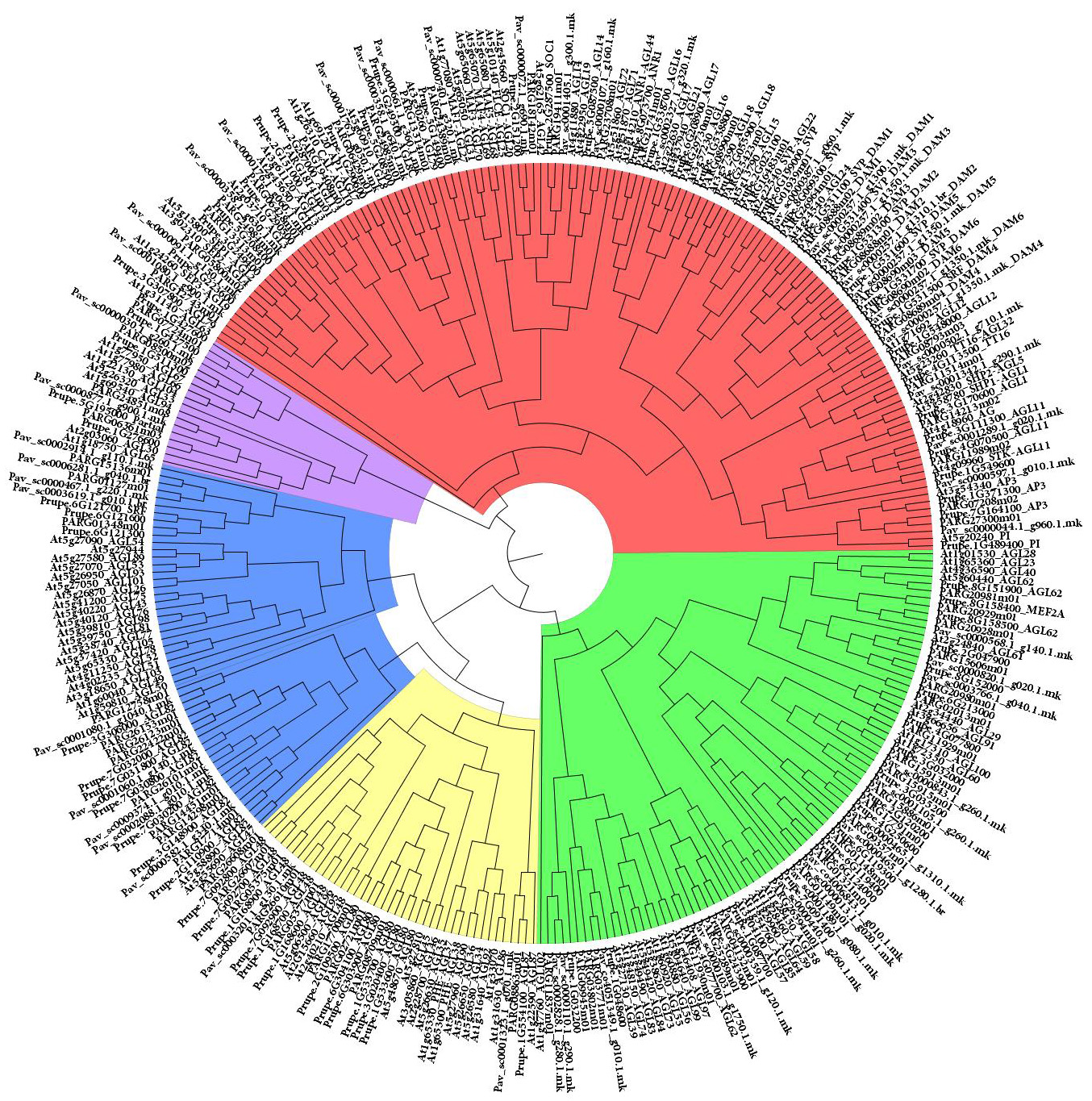


**Supplemental Figure 8**

Phylogenetic analysis of MADS-box genes belonging to the five groups Mα (green), Mβ (blue), Mγ (yellow), Mδ (violet), and MIKC (red). The full set of 327 identified MADS-box proteins (Supplemental file 1) were aligned ClustalX used to create a Neighbor-joining phylogeny tree in MEGAX with 100 bootstraps. Both ClustalX guide tree and MEGA NJ tree unambiguously separated Arabidopsis and peach M- and MYKC-type proteins (their classification was previously retrieved from the TAIR Gene family database and the Plant Transcription Factor Database, see Supplemental Material and Methods) into separated groups. Apricot and cherry MADS-box proteins were therefore classified using Arabidopsis and peach grouping as scaffold.

**SUPPLEMENTARY UPLOADED FILES**

**Supplemental Table 1**

Summary of *Prunus persica* (Ppe, peach) samples included in the original study (Yu et al., 2020) and those selected for the meta-analysis.

**Supplemental Table 2**

Summary of *Prunus avium* (Pav, cherry) samples included in the original study (Vimont et al., 2019)and those selected for the meta-analysis.

**Supplemental Table 3**

Summary of *Prunus armeniaca* (Par, apricot) samples included in the original study (Yu et al., 2020) and those selected for the meta-analysis.

**Supplemental Dataset 1**

Results of differential expression analyses in *Prunus persica* (Ppe, peach). Dataset includes gene expression values (FPKM) for all peach genes and the DEGs identified in each LRT test (HCR genotype, LCR genotype and Genotype x Dormancy interaction.

**Supplemental Dataset 2**

Results of differential expression analyses in *Prunus avium* (Pav, cherry). Dataset includes gene expression values (FPKM) for all cherry genes and the DEGs identified in each LRT test (HCR genotype, LCR genotype and Genotype x Dormancy interaction.

**Supplemental Dataset 3**

Results of differential expression analyses in *Prunus armeniaca* (Pav, apricot). Dataset includes gene expression values (FPKM) for all apricot genes and the DEGs identified in each LRT test (HCR genotype, LCR genotype and Genotype x Dormancy interaction.

**Supplemental file 1**

Amino acid sequences of Arabidopsis, peach, apricot and cherry MADS-box proteins used in this study.
